# Protective Effect of Pregnancy in Rural South Africa: Questioning the Concept of “Indirect Cause” of Maternal Death

**DOI:** 10.1371/journal.pone.0064414

**Published:** 2013-05-13

**Authors:** Michel Garenne, Kathleen Kahn, Mark Collinson, Xavier Gómez-Olivé, Stephen Tollman

**Affiliations:** 1 MRC/Wits Rural Public Health and Health Transitions Research Unit (Agincourt), School of Public Health, Faculty of Health Sciences, University of the Witwatersrand, Johannesburg, South Africa; 2 Institut Pasteur, Epidémiologie des Maladies Emergentes, Paris, France; 3 Institut de Recherche pour le Développement, UMI Résiliences, Centre Ile de France, Bondy, France; 4 Centre for Global Health Research, Umeå University, Umeå, Sweden; 5 INDEPTH Network, East Legon, Accra, Ghana; University of Vermont College of Medicine, United States of America

## Abstract

**Background:**

Measurement of the level and composition of maternal mortality depends on the definition used, with inconsistencies leading to inflated rates and invalid comparisons across settings. This study investigates the differences in risk of death for women in their reproductive years during and outside the maternal risk period (pregnancy, delivery, puerperium), focusing on specific causes of infectious, non-communicable and external causes of death after separating out direct obstetrical causes.

**Methods:**

Data on all deaths of women aged 15–49 years that occurred in the Agincourt sub-district between 1992 and 2010 were obtained from the Agincourt health and socio-demographic surveillance system (HDSS) located in rural South Africa. Causes of death were assessed using a validated verbal autopsy instrument. Analysis included 2170 deaths, of which 137 occurred during the maternal risk period.

**Findings:**

Overall, women had significantly lower mortality during the maternal risk period than outside it (age-standardized RR = 0.75; 95% CI = 0.63–0.89). This was true in most age groups with the exception of adolescents aged 15–19 years where the risk of death was higher. Mortality from most causes, other than obstetric causes, was lower during the maternal risk period except for malaria, cardiovascular diseases and violence where there were no differences. Lower mortality was significant for HIV/AIDS (RR = 0.29, P<0.0001), cancers (RR = 0.10, P<0.023), and accidents (RR = 0, P<0.0001).

**Interpretation:**

In this rural setting typical of much of Southern Africa, pregnancy was largely protective against the risk of death, most likely because of a strong selection effect amongst those women who conceived successfully. The concept of indirect cause of maternal death needs to be re-examined.

## Introduction

Maternal mortality focuses on the deaths of women that occur during the maternal risk period (MRP): pregnancy, delivery and 6-weeks post-partum. Causes of death of pregnant and delivering women are usually split into three categories: direct, indirect and fortuitous causes. According to the International Classification of Diseases 10^th^ revision (ICD-10), the “direct causes” are those resulting from obstetric complications of the pregnant state (pregnancy, labor, and puerperium), and from iatrogenic causes related to the pregnancy (interventions, omissions, incorrect treatment). The “indirect causes” are those resulting from previously existing diseases or diseases that developed during pregnancy which are aggravated by the physiological effects of pregnancy. The remainder, the “fortuitous causes”, are assumed to occur independent of the pregnancy [1]. The two key questions raised by these concepts are, firstly, how to define aggravation of the condition due to the pregnancy (would the woman have died the same way had she not been pregnant?); and secondly, the independence from other causes since the pregnancy induces a variety of changes in behavior (feeding, resting, risk taking) which could impact on many different causes of death.

The relationship between pregnancy and susceptibility to infectious diseases is complex. A few diseases are considered more lethal during pregnancy; the most striking is probably *listeria*, a bacterium which rarely kills outside of pregnancy except among diabetic and immuno-suppressed persons, but remains a rare cause of death [2]. Certain other conditions are believed to be enhanced by pregnancy including selected sexually transmitted diseases (syphilis, gonorrhea) and viral infections (influenza, viral hepatitis, measles, mumps, rubella), although with limited mortality impact in this age group [3]. The literature on malaria and pregnancy is controversial and has been reviewed recently in several papers [4]–[8]. In summary, it appears that women become more susceptible to *Plasmodium falciparum* malaria during pregnancy which may lead to cerebral malaria or induce severe anemia with multiple consequences (heart failure, post-partum hemorrhage). The effect of malaria on pregnancy seems more severe for very young women and during the first pregnancy. The effect of HIV/AIDS on pregnancy remains largely inconclusive [9]. Firstly, pregnancy does not accelerate the progression of HIV; and secondly, HIV infection reduces fertility. However, infection with HIV could have an effect on direct causes of death by inducing changes in behavior such as induced abortion or caesarean section (to prevent mother-to-child-transmission) which may increase maternal mortality. Furthermore, HIV treatment with anti-retroviral drugs (ARVs) may induce hypertension and liver disease [10]–[12]. Tuberculosis showed a similar pattern of not increasing risk during pregnancy [13]. The literature on cholera and other diarrheal diseases (e.g. shigella) in pregnancy is limited, sometimes conflicts and often concludes that there is no increased risk during pregnancy [14]. Thus, some acute infections that occur independently of a pregnancy (such as malaria and influenza) could be more severe during pregnancy, but this is not always the case. Certain chronic infections (such as HIV/AIDS and tuberculosis) do not appear to be more severe during pregnancy, and any apparent change in risk seems more likely associated with selection biases on biological or behavioral grounds.

The literature on anemia and pregnancy is as complex and controversial as that on infectious diseases [15]–[17]. Firstly, anemia is often a consequence of the pregnancy. Anemia in pregnancy can also be due to other causes such as infection with parasites and viruses (malaria, hookworm, HIV/AIDS), genetic factors and various haemoglobinopathies. There is no doubt that severe anemia can be a direct cause of maternal death through hemorrhage, cardiac failure and other maternal conditions.

In addition to naturally occurring diseases, iatrogenic causes and adverse effects of medical treatment may also increase the mortality risk of pregnant women. If the most common iatrogenic causes are well documented (caesarian section, induced abortion, toxic effect of certain drugs), new patterns are emerging with HAART (highly active anti-retroviral therapy). HAART was found to predispose to lipodystrophy, increase the risk of hypertension and be directly hepato-toxic in some cases, which could increase both direct and other causes of death of women in their reproductive years [10]–[12].

The literature on accidents and violence during pregnancy is limited. In the South African census, the maternal risk period was associated with somewhat higher mortality from accidents and violence: (RR = 1.32; 95% CI = 1.04–1.67). In Bangladesh, mortality from intentional and non-intentional injuries was higher during pregnancy [18] although this was not the case in Senegal [19]. The relationship between pregnancy and external causes of death is complex and most likely context-specific, with an attributable risk that is probably small even when the relative risk is greater than 1.

The aim of this study was to document the relative risk of death in women of reproductive age during and outside the maternal risk period by cause of death. Special attention was paid to selected infectious and parasitic diseases (HIV/AIDS, TB, malaria), non-communicable diseases (cancer, diabetes, cardiovascular diseases) and external causes (road traffic accidents, homicide, suicide), after separating out the direct obstetrical causes.

## Data and Methods

The study was conducted in the Agincourt sub-district of rural northeast South Africa, an area adjacent to southern Mozambique and under health and socio-demographic surveillance since 1992 [20]. Mortality from HIV/AIDS is very high yet accompanied by rising mortality from non-communicable diseases, cardiometabolic conditions in particular [21]–[22].

The Agincourt health and socio-demographic surveillance system (HDSS) includes full registration of vital events (births, deaths, in- and out- migrations) and prospective update of a range of individual and household attributes, in a population that today numbers some 90,000 people in 16,000 households [20]. The HDSS undertakes regular annual visits to all households, during which all deaths among persons resident in the population are recorded, irrespective of the place of death. So, the death of a woman that occurs in a nearby or more distant hospital after referral will be counted the same way as the death of a woman that occurs at home. Death registration by the HDSS is considered nearly complete, due to the experience of the field teams and sound community relations from the outset. Causes of death were investigated by systematic verbal autopsy (VA) conducted on deaths of all ages. VA questionnaires were reviewed by two independent physicians and, in cases of disagreement, were adjudicated by a third physician blind to the other findings [23]. VA diagnoses were validated by comparing with causes of death assessed in local hospitals [24]. Further details on methods and results can be found elsewhere [25]–[30].

Each death of women aged 15–49 years was further investigated with respect to her pregnancy status. This was done in two steps: in the comprehensive annual household survey update (with administration of an ‘individual death’ form) a question was systematically asked whether the deceased woman had died while pregnant or during delivery, or had delivered less than 6 weeks before her death. A subsequent check was conducted during the VA enquiry, with further details obtained on the duration of pregnancy and time since delivery.

Causes of maternal death were coded according to the ICD-10 classification. Causes in the O00–O95 list were considered as due to “obstetrical” causes, that is to the direct effect of pregnancy. There were no late obstetrical deaths (O96–O97) in the sample. Other causes of death were coded according to the underlying cause, whether due to infectious and parasitic diseases, to non-communicable diseases, to external causes, or were undetermined by the VA instrument. Although more detail is available, given small numbers results are presented for the most common causes of death among young women, specifically HIV/AIDS, pulmonary tuberculosis (TB), malaria and other infectious and parasitic diseases; cancers, cardiovascular and other non-communicable diseases; accidents, homicide and suicide; and undetermined causes.

Conceptually, the principle was to compute the relative risk (RR) of death during and outside the maternal risk period. A value of RR>1 indicated an increased risk of death consequent on pregnancy; and RR<1 a protective effect of pregnancy. The maternal risk period (MRP) was defined as 46 weeks (that is, 40 weeks of pregnancy, the delivery and 6 weeks post-partum). The principle of these calculations is presented in detail elsewhere [31]–[32]. We used the formula:
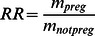
where m_preg_ is the death rate *during* the maternal risk period, and m_notpreg_ is the death rate *outside* the maternal risk period. The same calculations were carried out for selected causes of death, for particular age groups, and for age-standardized death rates. Age-standardized death rates were calculated using the World Health Organization Population Standard for low and middle-income countries [33].

Note that calculation of the RR required only two items of information: age specific fertility rates which determine the proportion of time spent in the maternal risk period; and the proportion of deaths that occurred during the maternal risk period. The formula applied was thus:
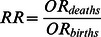
where OR_deaths_ is the odds-ratio of the proportion of deaths occurring during the maternal risk period; and OR_births_ is the odds-ratio of the proportion of time spent during the maternal risk period, defined as the product of fertility rate by the length of the MRP (46/52 weeks).

## Results

The Agincourt HDSS recorded 11,081 deaths of all ages from July 1992 to July 2010, of which 2170 deaths were of women aged 15–49 years. The average total fertility rate (TFR) over the period was 3.0 children per woman, a relatively low value for sub-Saharan Africa but average for rural South Africa over this period. The average probability of dying for women 15 to 49 years was 200 per 1000, a very high value (one woman in five died between age 15 and 49 years). The age pattern of fertility was characterized by relatively high levels among younger women, in particular adolescents 15–19 years. The age pattern of mortality also reflected high levels among young women, along with a flat pattern of mortality rates from age 30 to 49 years, a feature of high HIV/AIDS mortality environments. (Table 1).

**Table 1:** Age specific fertility, mortality, and pregnancy related deaths, Agincourt, South Africa, 1992–2010.

Age groupin years	PopulationPerson-years	Fertilityrate	Mortalityrate	% deathspregnancyrelated	Relative risk of death MRP/Other			
					RR	95% CI	P-value	Signif
15–19	75523	0.1020	0.0015	17.0	2.07	1.19–3.61	0.009	*
20–24	66082	0.1257	0.0037	11.0	0.98	0.64–1.52	0.938	
25–29	54461	0.1225	0.0074	12.1	1.13	0.82–1.56	0.443	
30–34	44256	0.1092	0.0101	7.8	0.80	0.55–1.15	0.225	
35–39	36326	0.0833	0.0108	5.2	0.69	0.43–1.12	0.130	
40–44	29184	0.0416	0.0107	3.3	0.90	0.46–1.74	0.747	
45–49	22696	0.0142	0.0114	0.0	0.00		0.000	*
Total	328528	0.0976	0.0066	7.4	0.84	0.71–1.00	0.051	*
Age-standardized					0.75		0.001	*

NB. MRP = maternal risk period; Fertility rate = births per 1000 women of given age per year; death rate = deaths per 1000 women of given age per year.

(*) P<0.05.

Overall, mortality from all causes combined was lower during the MRP than outside the period. The age-standardized relative risk was 0.75 (95% CI = 0.63–0.90; P = 0.001). Mortality during the MRP was lower than outside it for most age groups, except for adolescents 15–19 years where it was significantly higher (RR = 2.07; 95% CI = 1.19–3.61; P = 0.009). This excess mortality at age 15–19 was due entirely to eight deaths due to obstetrical causes among 15 deaths that occurred during the MRP. For causes other than obstetrical, the RR was no greater than one in this age group, and below one for some causes.

Overall, the relative risk of dying during the maternal risk period did not exceed one for any cause investigated apart from obstetrical causes. (Table 2) The relative risk was not significantly different from one for malaria, cardiovascular diseases and violence. For all other causes, the RR was less than one, and in many cases the difference was statistically significant: HIV/AIDS and TB (RR = 0.29, 95% CI = 0.21–0.41,P<10^−10^); other infectious and parasitic diseases (RR = 0.22, 95% CI = 0.03–1.63, P = 0.14); cancer (RR = 0.10, 95% CI = 0.01–0.73, P = 0.23); other non-communicable diseases (RR = 0.57, 95% CI = 0.27–1.23; P = 0.15); accidents (RR = 0; P = 10^−7^); unknown causes (RR = 0.58, 95%IC = 0.37–0.93, P = 0.02). Excluding obstetrical causes, the relative risk of death from all other and undetermined causes was RR = 0.41 (95% CI = 0.32–0.51, P<10^−10^), which shows the strong protective effect of pregnancy in this population. For causes other than obstetrical conditions, HIV/AIDS and TB, the relative risk was still well below one (RR = 0.53; 95% CI = 0.34–0.82; P = 0.004).

**Table 2:** Age standardized death rates and relative risk from selected causes, Agincourt, South Africa, 1992–2010.

	No. of deaths		Age standardized death rate				
Cause	MRP	Outside	MRP	Outside	RR	95%CI	Signif
Obstetrical	60	0	0.00227	0.00000			*
HIV/TB	37	987	0.00111	0.00378	0.29	0.21–0.41	*
Malaria	4	29	0.00012	0.00011	1.10	0.39–3.13	
Other infectious	1	29	0.00003	0.00012	0.22	0.03–1.63	
Cancer	1	77	0.00003	0.00034	0.10	0.01–0.73	*
Cardiovascular	5	78	0.00031	0.00033	0.94	0.38–2.32	
Other non-communicable	7	122	0.00026	0.00044	0.57	0.27–1.23	
Accidents	0	32	0.00000	0.00012	0.00		*
Violence	3	32	0.00008	0.00011	0.72	0.22–2.36	
Undetermined	19	337	0.00075	0.00129	0.58	0.37–0.93	*
Total	137	1 723	0.00495	0.00663	0.75	0.63–0.89	*
Non obstetrical	77	1 723	0.00269	0.00663	0.41	0.32–0.51	*

(*) P<0.05; MRP = maternal risk period.

ICD-10 codes: Obstetrical = O00–O95; Non obstetrical = any other cause. Cancer = C00–D49; Cardiovascular = I00–I67; Other non-communicable = any other D50–Q99 except cancer and cardio-vascular.

## Discussion

Overall, the Agincourt study shows that in rural South Africa, and potentially Southern African settings more widely, pregnancy is not a risk factor for most diseases other than obstetrical conditions. Indeed, the selection in favor of healthy pregnant women was so strong that, for most causes of death, mortality during the maternal risk period was about half of that outside the MRP. This effect remains poorly documented elsewhere, making it difficult to compare the Agincourt findings with other situations.

This finding raises questions on the definition and validity of “indirect cause” concept [34]. If there is no increased risk, and therefore no attributable risk arising from the pregnancy, is it possible for other conditions to be an “indirect cause” of maternal death? This issue has major implications for the measurement of maternal mortality in situations where HIV/AIDS is highly prevalent. If most causes other than obstetrical causes have little to do with the pregnancy, why include them when computing maternal mortality? This serves only to inflate the maternal mortality ratio (MMR), is misleading when comparing data over time and space, and can seriously bias reported trends in MMR. Would it not be a better reflection of reality to restrict the definition of maternal mortality to obstetrical causes of death?

There are a few conditions classified among the indirect causes known to be associated with pregnancy. Among infectious diseases, this is the case for instance of listeria, a rare disease for statistical purposes. Among chronic diseases, this is the case for instance of rheumatic heart disease, which may be exacerbated by pregnancy. More important, there are some violent deaths (homicide or suicide) which may be the consequence of pregnancy. These cases are important, but they require in-depth specific studies, and are unlikely to be properly captured in causes of death statistics of indirect causes. Categorizing these cases according to their ICD code, and coding independently the pregnancy status will be more informative for further statistical analysis. Analyzing them in specific studies, such as ‘Confidential Inquiries’ will also provide more useful information.

A similar argument could be made for HIV/AIDS and tuberculosis. Mortality from these conditions went up and down in Southern Africa, because of increasing HIV prevalence followed by widespread use of ARV treatment. Monitoring these changes is important for public health purposes, and coding separately whether women were pregnant or not is a valuable additional information. Monitoring toxic effects of ARV is also important. However, these points are beyond the classic coding of causes of death, and deserve further and specific research.

Selection in favor of healthy pregnant women can be easily understood. When women have a chronic infectious or non-communicable condition that may be terminal, they are less likely to become pregnant both for biological and behavioral reasons. As a result, women who are selected into partnership and pregnancy are less likely to die from these conditions. This adverse selection is more likely to apply to chronic conditions such as HIV/AIDS, TB, cancer, diabetes, hypertension and similar conditions. It is less likely to apply to acute conditions unless strong behavioral factors intervene, in which case the effect would probably be temporary.

The major causes of death among women 15–49 years were HIV/AIDS and TB accounting for 71% of determined causes for non-pregnant women. Selection for healthy pregnant women was therefore important in this population. We did not find comparable quantitative evidence elsewhere in the literature and this finding deserves further work. The lower risk of death from HIV/AIDS or TB remains plausible since the comparison is between women in the final stages of these diseases who die within a few months, and women who were healthy enough to become pregnant and therefore less likely to die within the maternal risk period. This does not mean that HIV/AIDS does not have any effect on the outcome of the pregnancy, whether through immunological and metabolic effects (such as anemia), or via treatment (ARVs may have an impact on hypertension and hepatic function). It simply means that the selection effect is much stronger and thus outweighs any deleterious effect.

The example of malaria deserves comment despite the small number of cases. Malaria can be an acute condition, determined by a mosquito bite and vector-borne transmission independent of the pregnancy. Here, one would expect the same mortality risk as for non-pregnant women. However, it can also be a chronic condition which can worsen because of the pregnancy by aggravating anemia. In this case one could expect an increased risk during pregnancy. More research on these effects is needed as the literature on this topic is controversial and the health outcome depends also on the local malaria profile (parasite strain, pattern of transmission), on treatments, and on parity. In Agincourt, the four malaria deaths among pregnant women occurred at younger ages (<35 years), whereas for non-pregnant women malaria deaths were more evenly spread (18 cases below age 35 years, 11 cases above). This pattern suggests an interaction between age, pregnancy and malaria, and in particular a higher risk associated with the first pregnancy, as found elsewhere [4]–[8].

Selection for cancer-related death or mortality from diabetes seems to be of similar nature as that associated with HIV/AIDS. The only cancer death among pregnant women was a case of cervical cancer in a 33 year old. There was no death from diabetes among pregnant women, compared with 12 deaths among the non-pregnant. Among the five cardiovascular disease deaths in pregnant women, one could be related to the pregnancy: a 23-year old woman who died of rheumatic heart disease, a condition exacerbated in pregnancy.

Selection for accidental deaths was also strong, since no road traffic accident and no other accident was noted among pregnant women, whereas these causes accounted for 2.3% (n = 32) of known causes of death among non-pregnant women. This could however be an effect of sample size.

Among deaths from violent causes, there was one suicide of a 19-year old which might have been related to the pregnancy. Of the two homicides, one seemed independent, a 16-year old killed by “bodily force”; and the other case, in a 30-year old woman, was unclear.

Returning to the coding of cause of death, we conclude that keeping focus on readily identifiable obstetrical causes as the underlying cause is very likely the most accurate and meaningful strategy for characterizing maternal deaths, especially in high HIV/AIDS environments. This will permit more valid comparisons across different settings and eliminate the need for assumptions on the proportion of HIV/AIDS deaths during the maternal risk period that contribute to indirect causes. Other causes are better coded according to their ICD-10 category, whether infectious, non-communicable or external, and with their pregnancy status coded separately. Further investigation will then be needed to assess whether these non-obstetric deaths occurring during the maternal risk period are potentially associated with the pregnancy.

This paper raises questions on the definition and validity of the concept of “indirect cause” and argues that including causes other than obstetrical causes may inflate the maternal mortality ratio and bias comparisons over time and space. Our analysis suggests that limiting the current definition of maternal mortality to the more specific obstetric causes would provide a better focus on Safe Motherhood, defined by the risk associated with pregnancy.

Our perspective was focused on medical statistics and epidemiology. A clinician’s perspective will, of course, be different, as they treat any condition experienced by pregnant or delivering women, irrespective of alternate international classification systems of direct or indirect ‘disease or cause of death’.

## References

[1] World Health Organization (1992) ICD-10: International Statistical Classification of Diseases and Related Health Problems, Tenth Revision. Geneva, World Health Organization.

[2] Silver HM (1998) Listeriosis during pregnancy. Obstet Gynecol Survey 53(12): 737–740. 10.1097/00006254-199812000-00004 9870235

[3] Laibl VR, Sheffield JS (2005) Influenza and pneumonia in pregnancy. Clin Perinatol 32(3): 727–738. 16085029 10.1016/j.clp.2005.04.009 PMC7119023

[4] Menendez C (2006) Malaria during pregnancy. Curr Molec Med 6: 269–273. 16515517 10.2174/156652406776055186

[5] Menendez C, Romagosa C, Ismail MR, Carrilho C, Saute F, et al (2008) An autopsy study of maternal mortality in Mozambique: The contribution of infectious diseases. PLoS Medicine 5(2): e44 doi: 10.1371/journal.pmed.0050044. 18288887 10.1371/journal.pmed.0050044 PMC2245982

[6] Uneke CJ (2007) Impact of placental Plasmodium falciparum malaria on pregnancy and perinatal outcome in sub-Saharan Africa: I: Introduction to placental malaria. Yale J Biol Med 80(2): 39–50. 18160989 PMC2140183

[7] Uneke CJ (2007) Impact of placental Plasmodium falciparum malaria on pregnancy and perinatal outcome in sub-Saharan Africa: II: Effects of placental malaria on perinatal outcome; malaria and HIV. Yale J Biol Med 80(3): 95–103. 18299721 PMC2248298

[8] Uneke CJ (2008) Impact of placental Plasmodium falciparum malaria on pregnancy and perinatal outcome in sub-Saharan Africa: III: placental malaria, maternal health, and public health. Yale J Biol Med 81(1): 1–7. 18604306 PMC2442721

[9] McIntyre J (2003) Mothers infected with HIV. Br Med Bull 67: 127–135. 14711759 10.1093/bmb/ldg012

[10] Suy A, Martínez E, Coll O, Lonca M, Palacio M, et al (2006) Increased risk of pre-eclampsia and fetal death in HIV-infected pregnant women receiving highly active antiretroviral therapy. AIDS 20(1): 59–66. 16327320 10.1097/01.aids.0000198090.70325.bd

[11] Mocroft A, Soriano V, Rockstroh J, Reiss P, Kirk O, et al (2005) Is there evidence for an increase in the death rate from liver-related disease in patients with HIV? AIDS 19(18): 2117–25. 16284461 10.1097/01.aids.0000194799.43799.ea

[12] Watts DH (2006) Treating HIV during pregnancy: an update on safety issues. Drug Saf 29(6): 467–490. 16752931 10.2165/00002018-200629060-00002

[13] Mnyani CN, McIntyre JA (2011) Tuberculosis in pregnancy. BJOG Int J Gynaecol Obstet 118(2): 226–231. 10.1111/j.1471-0528.2010.02771.x 21083862

[14] Ayangade O (1981) The significance of cholera outbreak in the prognosis of pregnancy. Int J Gynaecol Obstet 19(5): 403–407. 6120112 10.1016/0020-7292(81)90025-4

[15] Fleming AF (1989) Tropical obstetrics and gynaecology. 1. Anaemia in pregnancy in tropical Africa. Trans R Soc Trop Med Hyg 83(4): 441–8. 2694476 10.1016/0035-9203(89)90241-1

[16] Van den Broek N (1996) The aetiology of anaemia in pregnancy in West Africa. Trop Doct 26(1): 5–7. 8693577 10.1177/004947559602600103

[17] Williams MD, Wheby MS (1992) Anemia in pregnancy. Med Clin North Am 76(3): 631–647. 1578961 10.1016/s0025-7125(16)30344-3

[18] Khlat M, Ronsmans C (2000) Deaths attributable to childbearing in Matlab, Bangladesh: indirect causes of maternal mortality questioned. Am J Epidemiol 151(3): 300–306. 10670555 10.1093/oxfordjournals.aje.a010206

[19] Ronsmans C, Khlat M, Ba M, De Bernis L, Etard JF (2001) Evidence for a ‘healthy pregnant woman effect’ in Niakhar, Senegal. Int J Epidemiol 30(3): 467–473. 11416066 10.1093/ije/30.3.467

[20] Kahn K, Collinson MA, Gómez-Olivé FX, Mokoena O, Twine R, et al (2012) The Agincourt Health and socio-Demographic Surveillance System (Agincourt HDSS). Int J Epidemiol 41: 988–1001. 22933647 10.1093/ije/dys115 PMC3429877

[21] Tollman SM, Kahn K, Sartorius B, Collinson MA, Clark SJ, et al (2008) Implications of mortality transition for primary health care: a population-based surveillance study. The Lancet 372: 893–901. 10.1016/S0140-6736(08)61399-9 PMC2602585 18790312

[22] Kahn K, Garenne M, Collison M, Tollman SM (2007) Mortality trends in a new South Africa: Hard to make a fresh start. Scand J Public Health (suppl 69): 26–34. 10.1080/14034950701355668 PMC2825807 17676500

[23] Kahn K, Tollman SM, Garenne M, Gear JS (1999) Who dies from what? Determining cause of death in South Africa’s rural Northeast. Trop Med Int Health 4(6): 433–441. 10444319 10.1046/j.1365-3156.1999.00415.x

[24] Kahn K, Tollman SM, Garenne M, Gear JSS (2000) Validation and application of verbal autopsies in a rural area of South Africa. Trop Med Int Health 5(11): 824–831. 11123832 10.1046/j.1365-3156.2000.00638.x

[25] Kahn K, Tollman SM, Collinson MA, Clark SJ, Twine R, et al.. (2007) Research into health, population, and social transitions in rural South Africa: Data and methods of the Agincourt Health and Demographic Surveillance System. Scand J Public Health (suppl 69): 8–20. 10.1080/14034950701505031 PMC2826136 17676498

[26] Tollman SM, Kahn K, Garenne M, Gear JSS (1997) Reversal in mortality trends: evidence from the Agincourt field site, South Africa, 1992–1995. AIDS 13(9): 1091–1097. 10.1097/00002030-199906180-00013 10397540

[27] Tollman SM, Kahn K (2007) Health, population and social transitions in rural South Africa. Scand J Public Health (suppl 69): 4–7. 10.1080/14034950701525906 PMC2830106 17676497

[28] Garenne M, Kahn K, Tollman S, Gear J (2000) Causes of death in a rural area of South Africa: an international perspective. J Trop Pediatr 46(3): 183–190. 10893926 10.1093/tropej/46.3.183

[29] Weiner R, Tollman S, Kahn K, Penn-Kekana L (2007) Health and demographic surveillance sites contribute population-based data on maternal deaths in rural areas. South Afr Med J 97(10): 944–945. PMC2825809 18000573

[30] Zwang J, Garenne M, Kahn K, Collinson M, Tollman SM (2007) Trends in mortality from pulmonary tuberculosis and HIV/AIDS co-infection in rural South Africa (Agincourt). Trans R Soc Trop Med Hyg 101(9): 893–898. 17597174 10.1016/j.trstmh.2007.04.023

[31] Garenne M (2011) Estimating obstetric mortality from pregnancy related deaths (2011) Stud Fam Plann. 42(4): 237–246. 10.1111/j.1728-4465.2011.00287.x 22292243

[32] Stecklov G (1995) Maternal Mortality Estimation: Separating Pregnancy-Related and Non- Pregnancy-Related Risks. Stud Fam Plann 26(1): 33–38. 7785066

[33] Ahmad OB, Boschi-Pinto C, Lopez AD, Murray CJL, Lozano R, et al.. (2001) Age standardization of rates: a new WHO standard. Geneva, WHO, EIP/GPE/EBD, Discussion paper No 31.

[34] Cross S, Bell JS, Graham WJ (2010) What you count is what you target: the implications of maternal death classification for tracking progress towards reducing maternal mortality in developing countries. Bull World Health Organ 88: 147–153. 20428372 10.2471/BLT.09.063537 PMC2814479

